# An Effective Method of Isolating Honey Proteins

**DOI:** 10.3390/molecules24132399

**Published:** 2019-06-29

**Authors:** Aleksandra Bocian, Justyna Buczkowicz, Marcin Jaromin, Konrad Kamil Hus, Jaroslav Legáth

**Affiliations:** 1 Faculty of Chemistry, Rzeszow University of Technology, 35-959 Rzeszów, Poland; 2 Department of Pharmacology and Toxicology, University of Veterinary Medicine and Pharmacy, Komenského 73, 041 81 Kosice, Slovakia

**Keywords:** honey, proteins, phenol extraction, electrophoresis

## Abstract

Honey is a natural sweetener composed mostly of sugars, but it contains also pollen grains, proteins, free amino acids, and minerals. The amounts and proportions of these components depend on the honey type and bee species. Despite the low content of honey protein, they are becoming a popular study object, and have recently been used as markers of the authenticity and quality of honey. Currently, the most popular methods of protein isolation from honey are dialysis against distilled water, lyophilization of dialysate, or various precipitation protocols. In this work, we propose a new method based on saturated phenol. We tested it on three popular polish honey types and we proved its compatibility with both 1D and 2D polyacrylamide gel electrophoresis (PAGE) and MS (mass spectrometry) techniques. The elaborated technique is also potentially less expensive and less time-consuming than other previously described methods, while being equally effective.

## 1. Introduction

Honey is a natural product manufactured by honeybees from flower nectar or honeydew. It is a natural sweetener, as it is mostly composed of sugars: glucose, fructose, sucrose, and maltose; it also contains pollen grains, proteins, free amino acids, vitamins, flavors, minerals, and volatile compounds [1,2,3]. The amounts and proportions of these components are affected by various factors. These factors may be classified as: natural—such as type of plant and geographic region—and industrial—such as storage period [1].

Differences in protein composition of honey may be the result of different bee origin and flower nectar used by them [1,4]. Of interest are proteins originating from bees, which are secreted from the salivary and hypopharyngeal glands. The most abundant among them are major royal jelly proteins [4,5,6,7] but there are also present enzymes such as α- and β- glucosidase (invertase), α- and β- amylase (diastase), glucose oxidase, and others [3]. The proteins present in honey are widely used as markers of honey authenticity and adulteration tries [6,8] and also as a quality indicator [8,9]. Nevertheless, generally, this proteome is still very poorly researched, mostly because of the low amount of protein in honey (0.1–0.5%) [4]. That is the reason why analyses of the honey protein remains a challenge, and thus an efficient isolation method relating to sample (protein) concentration is desirable. The perfect method should not only be the most efficient and extract the highest possible number of proteins, but it should also be compatible with further gel electrophoresis and MS analysis [9].

Currently, the most popular method of protein isolation from honey is dialysis against distilled water and lyophilization of dialysate [5,6,10,11,12]. This method removes low-molecular-weight and interfering compounds, like sugars from proteins, by passive but selective diffusion through a semipermeable membrane. There are also a few reports about the chemical methods used to isolate honey proteins namely sodium tungstate [13], ammonium sulfate [14,15,16], and acetone precipitation [17]. There are also reports where authors use the combination of protein dialysis and precipitation [7]. A relatively small number of scientific reports describing protein isolation from honey prompted us to look for a new, effective method, which could be compatible with PAGE and MS techniques and would be less expensive and/or less time-consuming than those described before. That is why we have developed a new extraction method with the use of saturated phenol, tested it on three Polish honey types, and compared with the most frequently used methods. 

## 2. Results

**Figure 1:**
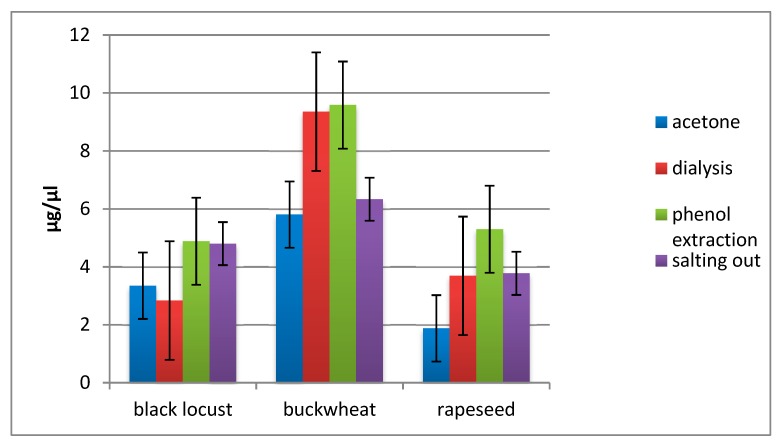
Protein concentration in extracted samples of three Polish honey types after applying four methods of isolation. Measurement performed with the use of *2D Quant Kit* in two technical repeats for each of three biological repeats.

Protein concentration measurement using the commercial kit revealed some differences in process efficiency (Figure 1). 

In order to determine statistically significant factors, a two-way analysis of variance was performed. The results presented in Table 1 show that both the method and the type of honey have an influence on the protein concentration. We can also see the influence of the interaction of the two traits on the variability of concentration.

**Figure 2:**
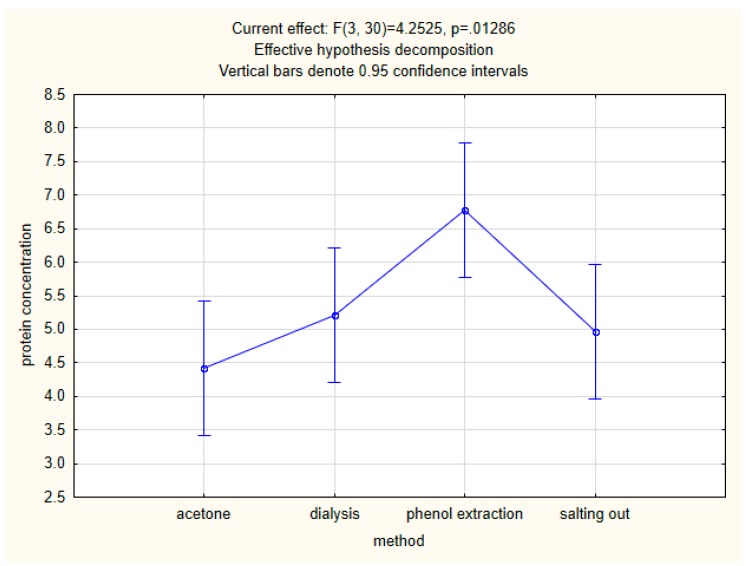
One-way analysis of variance indicating high efficiency of the phenolic method.

After a one-way analysis of variance, the statistical significance of differences between mean protein concentrations was confirmed. After the post-hoc test, we can conclude that in the case of the method only the phenolic method shows significant differences from the others (Figure 2).

**Figure 3:**
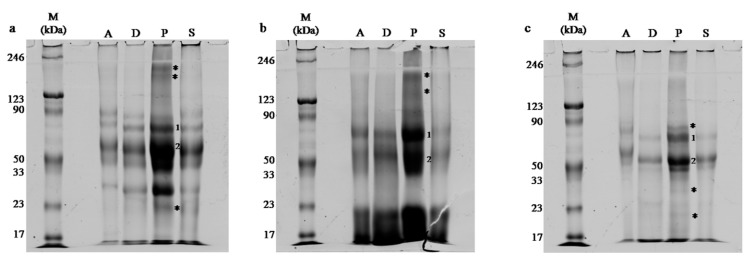
Gels obtained after SDS-PAGE electrophoresis of the: (**a**) black locust honey, (**b**) buckwheat honey, (**c**) rapeseed honey; 20 μg of protein were loaded on each lane. A—precipitation with acetone, D—dialysis with further lyophilization, P—extraction with saturated phenol, S—salting out with the use of ammonium sulfate; M—protein molecular mass marker Roti^®^-Mark PRESTAINED; *—detected additional bands; 1–2—proteins identified using MS, numbers correspond with Table 2.

A series of electrophoretic separations were performed for every honey variety and isolation method. Obtained protein maps clearly show that the honey proteome varies depending on the type of honey (Figure 3). Especially for buckwheat honey, the low molecular weight fraction is distinctive. The main goal of this experiment was to compare the efficiency of different protein isolation methods. In the case of black locust honey (Figure 3a), the band pattern visible on the obtained gels is almost identical for every extraction method. It can be also noticed that in the case of phenol extraction, bands are more intense as well as three additional bands corresponding with molecular weights of about 200 kDa and 23 kDa are visible, and marked with asterisks (Figure 3a). 

For buckwheat honey (Figure 3b) all obtained lanes are blurry but our experience shows that it is typical for this type of honey (data not presented). It is also clearly visible that the bands pattern distinctly differs from the other two analyzed honey types. Highly intense bands in lower mass areas are the hallmark of this honey. Despite the fact that an equal amount of protein was loaded on the gel, the most intense bands were observed in the lane with the extract obtained using phenol extraction. The band referring to 33 kDa is significantly more intense in the third lane than in others and it cannot be ruled out that there is more than one single band in this place. There are also two bands visible only in this sample, both with the molecular weight of more than 150 kDa (Figure 3b). 

In the third analyzed honey, the bands are also the most intense in the lanes with phenol extract (Figure 3c). It also appears that the band pattern is more similar to black locust honey than buckwheat, but in general, this gel has the lowest intensity. Three additional bands in the third lane could also be observed: one corresponding to 80 kDa and the other ones to 30 kDa and 20 kDa. There is also one additional band in the area between 20 kDa and 30 kDa present in the second lane (D).

**Figure 4:**
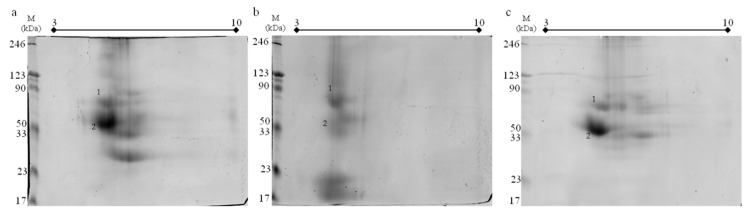
Gels obtained after 2D-PAGE electrophoresis of the: (**a**) black locust honey, (**b**) buckwheat honey, (**c**) rapeseed honey; 160 µg of protein obtained from phenol extraction were loaded on each pH 3–10 IPG strip; M—protein molecular mass marker Roti^®^-Mark PRESTAINED; 1-2—proteins identified using MS, numbers correspond with Table 2.

We have also performed 2D electrophoresis to separate proteins from each honey extracted with the use of saturated phenol (Figure 4). The goal here was to check the compatibility between the new extraction method and the standard protocol for two-dimensional electrophoresis. The results confirmed that our novel method can be easily utilized with electrophoretic techniques. The spots on the gels are similarly distributed in comparison with 1D separation. Characteristic spots for each honey are present on the gels, for example, low molecular weight proteins for buckwheat honey and the intense region near 23 kDa for black locust honey. There were also no problems with the subsequent spectrometric analysis.

Two dimensional protein maps obtained from phenol extracts are presented in the Figure 4. 

Two of the most intense bands present in all three honey types after phenol extraction were identified using MALDI ToF/ToF mass spectrometers. Results are summarized in Table 2.

## 3. Discussion

Honey quality control is important for two main reasons. First, changes in honey composition during storage stage may be observed. They are the result of chemical reactions: fermentation, oxidation and thermal processing [18]. Second, honey is a relatively expensive food product which causes it to be the object of numerous adulterations. That is why the testing and maintaining honey parameters may be useful for detecting possible adulterations, as well as for confirming proper conditions for the manipulation and the storage of honey [2]. Honey quality control is based on guidelines of the Codex Alimentarius which describes requirements standardizing the product processing, unified conditions and full transparency in its development and marketing. These regulations describe sugar and 5-hydroxymethylfurfural (HMF) content, acidity, diastase activity, and moisture as the maturity indicators, and as purity control parameters: ash content, electrical conductivity and insoluble solids in water [19]. However, recently also protein content and variability are seen as another parameter for quality [15,20], origin [12,21] and authenticity [6,8]. Increasing interest in honey proteins seems to be a very important factor leading to the improvement of the honey protein analysis methodology. 

We have tested four different protein isolation techniques. Dialysis with further lyophilization as a golden standard [5,6,10,11,12], two precipitation techniques: with ammonium sulfate [14,15,16] and modified using acetone [17] as well as extraction using saturated phenol. The latter, originally proposed by us, gave very satisfactory and promising results. 

This technique is based on the method developed by Hurkman and Tanaka in 1986 [22]. This technique was widely used mostly for plant material experiments such as leaves [23,24,25], roots [26,27], seeds [28,29], endosperm [30], pollen [31,32], stigma [33], and fruits [34,35]. However, it was also developed in analyses of fungi [36,37] and bacteria [38,39,40] proteomes, or even mammal cell cultures [41] and tissues [42]. Interestingly, it was proven that it is possible to adopt phenol extraction in food quality control, for example for analysis of proteins in wine [43], flour [44] mushrooms [45], or mussels [46]. 

The original method is based on the extraction buffer containing 0.7 M of sucrose [22] but in the case of honey, we hypothesized that natural high sugars content is sufficient to reverse phase system during centrifugation. Indeed, the phenol phase containing extracted proteins was the upper phase in the tubes and on the base of this observation we established that there is no necessity of using any buffer. Obtained extracts were easily solvable in both Laemli electrophoretic buffer and 2D buffer, which make them compatible with both electrophoretic methods. 

There are also no problems with protein identification using MS techniques but this is more likely merit of gel dyeing procedure and not extraction itself. Identified proteins were chosen as a test for compatibility with MS and they were present in all three honey types. Both identified proteins were of bee origin, which explains why they were present in all honey types. Alpha-glucosidase (invertase) degrades sucrose to fructose and glucose, so in mature honey its level is low. The process of sucrose degradation takes place during the honey ripening, so the level of this enzyme could be a maturing process indicator [47]. Moreover, invertase is thermolabile, so its activity can also be used as heat damage or inadequate storage indicator [48,49]. The second identified protein was major royal jelly protein 1, one of the most abundant bee-origin proteins in honey. This protein is also proposed as a marker of honey authenticity and quality [8] and a few years ago even ELISA test using MRJP (Major Royal Jelly Protein) was developed for screening of honey adulteration based on the addition of industrial sugar syrups and/or of honey from bees fed with sucrose syrup [50].

Obtained one-dimensional protein maps (Figure 3) have sufficient resolution and quality and they do not deviate from recently published standards [6,7,8,15] and may even exceed them. We cannot explain exactly why all lanes, with proteins obtained after phenol extraction, are the most intense on gels (Figure 3). Possible explanations for this phenomenon could be that after phenol extraction, there are superior interactions in the sample between proteins and the dye which would cause more intense bands on gels. We have also concluded that it is possible that the phenol method yields in less number of additives in the sample. These additives may interfere with the protocol for the determination of protein concentration. Interfering substances in other extraction methods could interact with assay reagents leading to overestimation in total protein content. We performed some experiments with different protein concentration levels loaded on the gels and the results were the same (data not shown). Nevertheless, it is hard to consider this as a disadvantage, especially in the context of further protein identification using MS techniques where high protein content in band excised from gel makes identification easier. Moreover, if that was the case, the results from protein concentration measurements (Figure 1) could also be influenced by this effect. In such situations, this data would be falsely overstated. However wrong it may be, this would actually cause larger differences in protein yield between analyzed methods which in essence even enhances the final assessment of this new technique. Two-dimensional maps (Figure 4) are also satisfying in resolution and quality. There are only a few published works [17,48,49] with which we can compare our maps and on their basis, we can say that the goal of developing an effective and compatible method has been achieved.

The greatest advantage of this new method is the fact that statistical analysis has shown that this method is more efficient than other methods tested by us. Moreover, it is less time consuming than the golden standard (dialysis with further lyophilization) and reduces the time it takes to isolate protein from 32 to 12 h. Although the time needed for isolation is similar to acetone precipitation and longer than in salting out, the presence of additional bands in Figure 2 indicates that the isolation process can be more efficient. The described phenol method does not require expensive equipment such as lyophilizer or acquisition of dialyzers, which makes it an effective low-cost method. To summarize, in our opinion this new method based on saturated phenol extraction is worth closer attention, especially considering that protein profiles are one of the most important methods of tracing honey authenticity [50], gaining more and more importance and popularity. Our experience shows that honey types differ significantly in the amount of protein. That is why we have proposed a method that allows concentrating the sample by dissolving in a small volume of buffer the extract obtained from a large amount of honey, in the hope that it will be interesting for researchers who are struggling with this problem.

## 4. Materials and Methods 

Proteins were isolated from three most popular Polish types of honey: rapeseed, black locust, and buckwheat. Honeys were obtained from the official distribution of Beekeeping Organisation of Podkarpackie Voivodeship (south-eastern region of Poland).

All isolations were carried out with the same proportions: 1 g of honey was used as material and the final precipitate was dissolved in 500 µL of standard Laemli buffer. All experiments were performed in three biological repeats (three independent isolations). For this experiment, four methods were used: precipitation with acetone (A), dialysis with further lyophilization (D), extraction with saturated phenol (P) and salting out with the use of ammonium sulfate (S).

For precipitation with acetone (A), honey was diluted with deionized water in 1:1 proportion (*w*/*w*) and 5 volumes of pure acetone were added. After 12 h of precipitation in –4 °C, tubes were centrifuged for 30 min at 10,000 *g* and pellets were dried in the open air. For salting out (S), honey samples were five times diluted with deionized water (*w*/*w*) and precipitated on a magnetic stirrer with the use of solid ammonium sulfate up to the final concentration of 4 M. The samples were then centrifuged for 30 min at 10,000 *g*. Dialysis (D) was performed on honey samples diluted with deionized water in 1:1 proportion (*w*/*w*) in D-Tube™ Dialyzer Maxi (MWCO 3.5kDa, Merck Millipore, Burlington, MA, USA) for 24 h against deionized water, changed every 8 h. After that dialysates were lyophilized for 8 h.

Extraction with phenol (P) was performed with honey samples diluted with deionized water in 1:1 proportion (*w*/*w*). To this solution, one volume of saturated phenol was added (pH 7.9 Biotech Grade, PHE510, BioShop, Burlington, ON, Canada) and samples were intensively mixed in a vortex mixer for 15 min. The samples were then centrifuged for 30 min in 10,000 *g* and the upper phase was transferred to new tubes and precipitated overnight with five volumes of 0.1 M ammonium acetate in methanol. Before electrophoresis samples were centrifuged for 30 min in 20,000 *g* and dissolved as described above.

All samples after isolation were finally dissolved in 500 µL of standard Laemli buffer and protein concentration was measured using 2D Quant Kit (80-6483-56, GE Healthcare, Little Chalfont, UK) in two technical repeats with the BSA as a standard in accordance with the manufacturer’s instructions. 

Results were analyzed statistically by Statistica software v. 12.5. The protein concentration met the assumptions of normality and homogeneity of variance, so to assess the differences between the groups, a main effects analysis of variance and LSD (post-hoc) test were used.

SDS-PAGE was performed using Mini-Protean II apparatus (Bio-Rad Laboratories, Inc., Hercules, CA, USA) on 10% resolving gels (with 5% stacking gels) according to the method of Laemmli [51]. Samples were boiled for 5 min before electrophoresis and applied on gels in equal amounts 20 µg of proteins per lane. 

Phenol extracted proteins were also separated using 2D electrophoresis. Precipitant after centrifugation was dissolved in IEF (isoelectric focusing) buffer containing: 7 M urea, 2 M thiourea, 2% Nonidet P-40 substitute, 0.5% IPG buffer pH 3–10, 0.002% bromophenol blue, and 18 mM DTT. After protein concentration measurement (as described above) 160 µg of protein was loaded on ReadyStrip™ IPG Strips 7 cm, pH 3–10 (Bio-Rad Laboratories, Inc., Hercules, CA, USA). Parameters of isoelectric focusing were programmed according to the manufacturer’s instructions on the PROTEAN IEF Cell device (Bio-Rad Laboratories, Inc., Hercules, CA, USA). Before the second dimension the strips were equilibrated for 15 min in SDS equilibration buffer solutions (6 M urea, 75 mM TRIS-HCl pH 8.8, 29.3% glycerol, 2% SDS, 0.002% bromophenol blue), the first containing 1% DTT and the second 2.5% iodoacetamide instead of DTT. SDS-PAGE (second dimension) was performed on 10% PAA gels in Mini-Protean II apparatus (Bio-Rad Laboratories, Inc., Hercules, CA, USA) according to the method of Laemmli [51]. 

After electrophoresis, all gels were stained with colloidal Coomassie Brilliant Blue G-250 overnight and then washed for 24 h with deionized water in order to remove the remains of the dye [52].

Two of the most intense bands obtained on lanes from phenol extraction and present in all analyzed SDS-PAGE gels and corresponding spots from 2D gels were excised from the gels and digested using Sequencing Grade Modified Trypsin (V5111, Promega, Madison, WI, USA), according to a modified method adapted from Shevchenko et al. [53]. Proteins were identified on a MALDI ToF/ToF mass spectrometer (Autoflex Speed, Bruker Daltonics, Billerica, MA, USA) and detailed parameters of the analyses were described in our previous works [54,55]. 

## Figures and Tables

**Table 1:** Summary of two-way analysis of variance.

Effect	Univariate Tests of Significance for Protein Concentration				
	SS	DF	MS	F	*p*
intercept	1027.853	1	1027.853	474.8688	0.000000
Honey type	106.313	2	53.156	24.5583	0.000000
method	27.614	3	9.205	4.2525	0.012858

SS—the sum of squares; DF—the degrees of freedom; MS—mean square; F—the F-test, *p*—*p*-value (probability).

**Table 2:** The results of MS analysis performed on two selected proteins.

No ^1^	Identified Protein ^2^	Accession ^3^	Organism ^4^	Mass (kDa) ^5^	S ^6^	*m*/*z*^7^	Peptide Sequence ^8^
1	Alpha-glucosidase	Q17058	*Apis mellifera* (Honeybee)	65	87	1720.90	IYTHDIPETYNVVR
					55	1482.70	VDALPYICEDMR
					65	1395.58	EDLIVYQVYPR
					44	1188.30	DVLDEFPQPK
2	Major Royal Jelly Protein 1	O18330	*Apis mellifera* (Honeybee)	49	51	1615.83	IMNANVNELILNTR
					57	1398.41	FFDYDFGSDER
					63	1294.48	EALPHVPIFDR
					91	1747.87	MVNNDFNFDDVNFR

^1^ Spot number corresponding with Figure 2 and Figure 3; ^2^ Protein name in database; ^3^ Database (UniProt) accession number of homologous proteins; ^4^ Organism from which protein identification originates; ^5^ Molecular mass of protein; ^6^ Score parameter (Protein identification was performed using the Mascot search with probability based Mowse score. Ions score was—10 × log(P), where *p* was the probability that the observed match was a random event. Mascot defined thresholds which indicated identity or extensive homology (*p* < 0.05) was 48); ^7^ *m*/*z* of precursor ion (MH+); ^8^ Peptide sequence derived from LIFT analysis. Identification of proteins by MS/MS method was conducted by comparing obtained sequences with sequences from the database.
